# Complex and Conflicting Social Norms: Implications for Implementation of Future HIV Pre-Exposure Prophylaxis (PrEP) Interventions in Vancouver, Canada

**DOI:** 10.1371/journal.pone.0146513

**Published:** 2016-01-12

**Authors:** Rod Knight, Will Small, Anna Carson, Jean Shoveller

**Affiliations:** 1 Faculty of Health Sciences, Simon Fraser University, Burnaby, Canada; 2 British Columbia Centre for Excellence in HIV/AIDS, Vancouver, Canada; 3 School of Population and Public Health, University of British Columbia, Vancouver, Canada; The University of New South Wales, AUSTRALIA

## Abstract

**Background:**

HIV Pre-Exposure Prophylaxis (PrEP) has been found to be efficacious in preventing HIV acquisition among seronegative individuals in a variety of risk groups, including men who have sex with men and people who inject drugs. To date, however, it remains unclear how socio-cultural norms (e.g., attitudes towards HIV; social understandings regarding HIV risk practices) may influence the scalability of future PrEP interventions. The objective of this study is to assess how socio-cultural norms may influence the implementation and scalability of future HIV PrEP interventions in Vancouver, Canada.

**Methods:**

We conducted 50 interviews with young men (ages 18–24) with a variety of HIV risk behavioural profiles (e.g., young men who inject drugs; MSM). Interviews focused on participants’ experiences and perceptions with various HIV interventions and policies, including PrEP.

**Results:**

While awareness of PrEP was generally low, perceptions about the potential personal and public health gains associated with PrEP were interconnected with expressions of complex and sometimes conflicting social norms. Some accounts characterized PrEP as a convenient form of reliable protection against HIV, likening it to the female birth control pill. Other accounts cast PrEP as a means to facilitate ‘socially unacceptable’ behaviour (e.g., promiscuity). Stigmatizing rhetoric was used to position PrEP as a tool that could promote some groups’ proclivities to take ‘risks’.

**Conclusion:**

Stigma regarding ‘risky’ behaviour and PrEP should not be underestimated as a serious implementation challenge. Pre-implementation strategies that concomitantly aim to improve knowledge about PrEP, while addressing associated social prejudices, may be key to effective implementation and scale-up.

## Introduction

HIV Pre-Exposure Prophylaxis (PrEP) has been found to be efficacious in preventing HIV acquisition among seronegative individuals in a variety of risk groups, including men who have sex with men (MSM) [1,2], people who inject drugs [3] and serodiscordant heterosexual couples [4]. However, a growing body of research indicates acceptance and uptake of PrEP among key high-risk population subgroups remains low within and across many global contexts [5,6,7], including those that have yet to licence antiviral medication for PrEP [5,8]. There is some evidence that even within high-risk groups (e.g., MSM) many people do not personally feel at elevated risk for HIV acquisition and therefore do not view themselves as potential consumers of PrEP [9,10]. Previous research has also identified individual-level (e.g., HIV risk literacy; concerns about drug toxicity) [11] and structural-level factors (e.g., costs; stigmatizing or negative attitudes about PrEP by health care providers) as influencing the scalability of PrEP (e.g., rate and pace of uptake; equitable reach) [12]. Recent research from the United Kingdom demonstrates that some perceive PrEP as a controversial intervention with high potential to have unintentional, negative impacts [13]; studies in the United States also report concerns that PrEP interventions may unintentionally result in risk compensation behaviour [14]. To date, however, it remains unclear how socio-cultural norms (e.g., attitudes towards HIV; social understandings regarding HIV risk practices) may influence the scalability of future PrEP interventions in other contexts, including Canada.

Vancouver, Canada, offers an interesting context within which to examine factors influencing the scalability of future PrEP interventions, especially among young men who represent an important population subgroup in terms of HIV risk. Despite the success of HIV prevention efforts in reducing HIV incidence rates in other population subgroups (e.g., people who inject drugs) [15], HIV incidence rates among young men remain high within the current study context (compared to older men and women). Furthermore, in Vancouver, exploratory quantitative data also indicates low acceptability of PrEP among key risk groups of young men (e.g., those who inject drugs) [8]; this reflects research in other settings on young men’s views on PrEP [11] and their low levels of engagement with other components of the HIV continuum of care (e.g., HIV testing) [16, 17].

While there is no antiviral medication yet licensed for PrEP use in Canada [18], physicians can prescribe PrEP for “off-label” use [19]. Thus, we undertook the current study in order to examine socio-cultural norms about PrEP *prior* to the widespread use of PrEP within Vancouver. In particular, young MSM and men who inject drugs represent key priorities for PrEP initiation under future Health Canada guidelines. Using in-depth qualitative interviews, we gathered detailed descriptions from young men regarding their knowledge and perceptions about PrEP and how broader social norms, including social stigma regarding HIV and HIV risk behaviour, may influence its subsequent acceptance and uptake, if that was to occur.

## Methods

### Recruitment and data collection

This study received approval from the University of British Columbia’s Behavioural Research Ethics Board (UBC BREB, #H12-01936); participants provided their written informed consent. The UBC BREB approved the following protocol. Participants were recruited using posters at clinical (e.g., sexual health clinics) and non-clinical settings (e.g., community centers), as well as using online strategies (Facebook Ads; Craigslist). We also recruited from the At-Risk Youth Study (ARYS), a prospective cohort of young people with a history of having used illicit drugs (other than marijuana) and being street-entrenched [20]. Prospective participants were told that the study was being conducted to better understand young men’s perceptions of and experiences with various HIV-related interventions. Eligibility criteria included: identifies as a man; speaks and understands English; aged18-24; and resides in Metro Vancouver. Interviews occurred at our research offices and were conducted by co-authors RK and AC, two highly experienced interviewers. Participants were asked to describe what they know about PrEP and were subsequently provided with additional information. Participants were then asked to provide their perspectives regarding PrEP interventions if they were to unfold on a scaled-up level in Vancouver in the future. Each interview lasted approximately one hour and was audio-recorded. Participants received CDN$25 for completing an interview.

### Data Availability Statement

All relevant data are presented within the paper and are fully sufficient to replicate the study findings.

### Data analysis

Interviews were transcribed with identifiable information removed, checked for accuracy and uploaded to *QSR NVivo 10***™** for data analysis. We used constant comparative techniques [21] to develop an initial set of codes that featured participants’ understandings, perceptions and opinions regarding PrEP. As coding progressed, we organized our codes into ‘trees’ to group the open codes into more specific categories related to the objectives of this study, at which point we began to relate our data to the previous empirical and theoretical work in this area.

## Results

### Study participants

Of the 50 study participants, 16 identified as gay, bisexual, Two-Spirit and 24 had a history of using illicit drugs and experiences with being street-entrenched. See Table 1 for more details.

**Table 1:** Socio-demographic characteristics of study sample.

Age	*(n)*	(%)
18–19	5	10
20–21	14	28
22–23	25	50
24	6	12
**Ethnicity**		
Aboriginal	6	12
African-Canadian	1	2
Euro-Canadian	26	52
Latin	2	4
South East Asian	7	14
Middle Eastern	1	2
Other	7	14
**Living Arrangement**		
With parents	9	18
With friends or partner	22	44
Alone	7	14
In a shelter or on the street	11	22
In a recovery house	1	2
**Sexual Identity**		
Bisexual	8	16
Gay	7	14
Heterosexual/straight	34	68
Two-Spirit	1	2
**Gender Identity**		
Transgender man	1	2
Cisgender man	49	98
**Number of Times Tested**		
0	5	10
1	15	30
2+	30	60
**Recruitment Medium**		
Online advertising	23	46
Posters	2	4
ARYS	24	48
Other	1	2

#### (1) “One-a-day”–easy, convenient, effective

At the outset of each interview, most participants indicated that they did not know very much about PrEP or how it works. Thus, each interview began with us providing a standardized and brief overview of PrEP and how it functions. Initially, many participants likened PrEP to female birth control (“the pill”), frequently referring to the ease and convenience of being able to take a daily dose and be assured of protection. For example, one 22-year-old gay man who reported multiple concurrent sexual partners described:

*It’s just like girls can get birth control*, *you know what I mean? Like*, *if I can go and just say*, *like*, *“Don’t wanna get HIV” and like* [the doctor could say] *‘Here you go*. *Here’s your “one-a-day”‘*. (#023)

Most respondents indicated that PrEP was not for everyone, explaining that it would be best suited for use by ‘high-risk’ groups (e.g., sexually “promiscuous” people). Some respondents positioned PrEP use by ‘high-risk’ people as being an act of taking on responsibility for both personal and public health reasons. For example, a 23-year-old straight man explained his view that PrEP would be appropriate for gay men to use:

*I think that’s a great approach if they want to take it*, *by all means*, *they should be protecting themselves and potentially future partners that they have*. (#029)

Like this young man, many participants who did not perceive *themselves* to be at ‘high risk’ for HIV positively viewed PrEP’s convenient, ‘once-a-day’ approach and acknowledged its potential as an effective risk-reduction strategy for those *‘others’* who were at ‘high risk’.

#### (2) ‘Bullet-proof’–exacerbating risky practices

Some participants described how the introduction of PrEP might affect perceived HIV risk. For instance, one 20-year-old straight man who was engaged in sex work at the time of our interview worried that the expansion of PrEP as a risk-reduction strategy could result in a ‘shift’ in how ‘high-risk’ individuals understand HIV risk and, thus, exacerbate the likelihood that they would choose to engage in risky practices:

*I wouldn’t just hand PrEP out over the counter*, *like Advil*.***™*** […] *It’s not like it’s a cure*. *Remember*, *it’s a treatment*. *‘If it* [PrEP] *was given out like Skittles*, *everyone is going to think it’s a cure’* (#041).

Although clinical guidelines indicate that those who initiate PrEP are to be clinically monitored and tested for HIV and other STIs at recommended intervals, some participants were concerned that PrEP could inadvertently reduce some men’s engagement with HIV testing. As one 22-year-old bisexual man worried:

*I wonder how frequently they would still test*. *Because I think that frequent testing would actually be a more positive way of making sure that people are safe and cared for than treating somebody for a disease they don’t have*. (#017)

These kinds of statements reflect an overall conception of PrEP as the provision and consumption of a medication. In our sample, few people focused on the fact that PrEP approaches also involve ongoing clinical monitoring and engagement with care providers and PrEP was therefore generally characterized quite simplistically as ‘taking a pill’.

Others suggested that young men who decide to initiate PrEP might be more likely to engage in condomless sex, promiscuity, or other ‘high-risk’ behaviour. For example, one 20-year-old gay man who described himself as being ‘low-risk’ for HIV described how PrEP could unintentionally result in increased sexual activity among some individuals:

*If they know that ‘Okay*, *I’m on treatment*. *And because ‘I’m on treatment*, *I’m like*, *you know*, *I’m bulletproof*. *I could be even crazier!’ I don’t think it’s a good idea*. (#024)

Similarly, a sub-set of participants who had previous experiences with illicit drug injecting worried that those who choose to initiate PrEP may be less likely to seek out and use sterile syringes, as one 20-year-old straight man who was living on the streets at the time of our interview explained:

*They’d probably think that ‘Oh*, *I take this pill*, *so I’m not going to catch anything*.*’ And then they’re just gonna share needles*. (#034)

The participants in our study included people who could be characterized as ‘low risk’ (e.g., those who had not been sexually active for several months) as well as those who could be considered at ‘high risk’ for acquiring HIV (e.g., those who report frequent experiences of sharing syringes or who avoid the use of condoms). However, both ‘low’ *and* ‘high’ risk participants articulated the assertion that PrEP could exacerbate risk practices amongst so-called ‘high-risk’ individuals.

#### (3) ‘High-risk lifestyles’–stigma and individualized practices

Use of PrEP was often portrayed as being problematic because it was viewed as an ‘excuse’ from adherence to other risk-reduction practices and, therefore, it was also often viewed as contributing to ‘high-risk’ lifestyles. The language use and tone of these portrayals were highly individualizing and accusatory, as the following example from a 19-year-old straight man illustrates: Just because you are on the most amazing treatments in the world, I don’t think it excuses not having safe sex (#002). In expressing their concerns about PrEP, participants, including many who identified as gay, bisexual, or MSM, frequently drew on stereotypes that link promiscuity and condomless sex with a ‘gay lifestyle’ and used those prejudices to underpin their negative opinions about PrEP. For example, one 20-year-old gay participant who was involved with multiple concurrent sexual partners at the time of our interview described PrEP’s potential impact on gay men’s sex lives in nihilistic terms:

*I think it’s* [PrEP] *ridiculous*. *I think it’s this kind of idea that the drugs will fix it instead of being a little bit more safe* […] *It seems a little bit like a kind of a “Rolls Royce” kind of condom to me–it seems a little bit extreme*. […] *If people are paying thousands of dollars out of pocket so they can be having bareback sex*, *I think that’s a little bit insane*. […] *I think that if you’re offloading kind of the responsibility for safe sex onto a pill* […] *it has this kind of fatalism for me that I don’t appreciate*. (#014)

Participants’ concerns about PrEP, promiscuity, and condomless sex, especially amongst people who identify as gay, bi-sexual and MSM, were often juxtaposed against more conventional approaches to HIV prevention (condom use; limiting numbers of sex partners). As one 20-year-old gay participant who described himself as being ‘low-risk’ for HIV explained:

*I think the fine line is this*: *the people who are [already] promiscuous are going to be even more sexually active than they were before*, *and that defeats the purpose of having the antivirals*. *Either way*, *they’re probably gonna get it [HIV]*. (*#*024)

In contrast to these somewhat fatalistic perspectives, participants also described scenarios under which they thought PrEP would be an acceptable risk-reduction strategy. A frequent example of a condition under which PrEP would be viewed to be acceptable was among HIV serodiscordant couples. For example, one 21-year-old straight man (who himself reported having multiple concurrent sex partners) described how he thought PrEP would be highly acceptable and helpful for ‘faithful’ serodiscordant couples, while highly unacceptable for ‘promiscuous’, single people:

*In [the situation of serodiscordant couples]*, *I’d be like “Okay*, *cool*.*” Cause*, *they’re in a committed relationship*, *they’re being faithful*. *So*, *obviously they’re not gonna run around and be all promiscuous or running all over town*, *right? So that would make sense*, *cause you’re helping someone in a committed relationship to stay healthy*. *Whereas*, *on the flip side*, *you’re helping someone that’s promiscuous to keep being promiscuous*. (#009)

Thus, a complex and sometimes conflicting narrative emerged in which PrEP was described both as a ‘responsible’, safety-enhancing strategy (when used by monogamous serodiscordant couples) *or* a highly ‘irresponsible’ approach that excused people from engaging in other, more conventional risk-reduction practices. A 22-year-old straight participant who had previously engaged in same-sex sexual behaviour and reported a history of injection drug use went as far to say that he feared that being on PrEP could serve as a proxy for one’s stigmatized social status:

*It would almost turn into an insult*. *People would be like ‘Oh well he’s on the preventative treatment*. *That’s the sort of person he is*.*’ Or*, *‘You’re such a slut! You should be on preventative treatment’*. *And that would be a huge insult if such were the case*. (#007)

A strong theme emerged across the interviews whereby participants suggested that rather than choosing to initiate PrEP, individuals at high risk of HIV should focus their efforts on reducing or eliminating their risk through other practices (e.g., use condoms; stop sharing syringes). Clearly, these narratives position risk, risky behaviour and risk groups in highly contentious ways–and the absence of recognition of structural risk or other contextually based factors that contribute to risk of HIV was remarkable. The strong ways in which individualization of responsibility for reducing HIV risk is succinctly summarized in the following quote from one 24-year-old straight man that was not sexually active at the time of our interview, as he said:

*Instead of continuing these risky behaviours and using HIV medication*, *maybe people should just stop the behaviour*, *right? (#020)*.

Furthermore, several participants felt that those who choose to engage in “high-risk lifestyles” while using PrEP should be required to pay for the treatment themselves, rather than have government subsidies cover the costs. For example, one 21-year-old straight man that reported multiple concurrent sexual partners explained:

*Someone that’s living a–what would be classified a “high-risk lifestyle"*, *then obviously they need to deal with the consequences and take precautionary measures*. *I can’t say it’s wrong*, *I mean*, *they can do whatever the hell they wanna do*. *It’s their life*. *But with regards to providing treatment? They should*, *in my opinion*, *pay for it*. *Because they’re putting themselves in high-risk positions*. (#009)

## Discussion

Most of the study participants had very little previous knowledge about PrEP prior to enrolling in the current study. Yet, they frequently expressed negative views regarding PrEP, which we found surprising considering that the study included a relatively diverse sample of young men who resided in Vancouver–a city well-known as a Canadian, if not international, leader in providing universal HIV treatment and care. The vociferous expression of objections to PrEP identified by participants of both ‘low’ and ‘high’ HIV risk status (e.g., including those who would be eligible for PrEP under United States Centers for Disease Control and Prevention PrEP guidelines) is important for public health officials to take note of, given that young men have been identified as a priority population for PrEP scale up in British Columbia [22] and internationally [23]. As well, a large number of young men in our study expressed strong opinions about who should bear the cost of PrEP (the individual or the government)–though, the view that the individual should pay the total cost were largely expressed by those who were at low risk.

It is also worth considering the extent to which young men’s individually oriented concerns about PrEP are embedded within a responsibilization discourse (e.g., one that idealizes rational, contract-making individuals in a free-market society). Such a discourse emphasizes the role of the individual’s obligation to ‘do’ public health, thereby taking responsibility out of the realm of the State. For example, the tendency for participants to describe PrEP as being “a bit extreme” often took place as they reflected on the extremely high costs of PrEP, with less reflection on the broader reasons that the costs might remain high (e.g., lack of licensing; profit-driven interests of ‘big pharma’). In doing so, social and structural inequalities (e.g., ‘inflated’ price of PrEP; HIV stigma) were often reduced to individually related problems, rather than issues that might require State intervention. Indeed, as we have argued elsewhere, these discourses tend to situate agentic, rather than structural, influences as the morally relevant determinants of health and illness [24].

Understanding these types of normative influences within an implementation context is important for the uptake of any public health intervention [25,26,27,28], including PrEP [29]. The results of this study indicate that stigma associated with HIV and stigmatizing attitudes towards PrEP users may represent a serious implementation challenge in the Vancouver context. As these and previous findings [30, 31,32] underscore, pharmacotherapeutic approaches like PrEP are also inherently *social* interventions that require detailed understandings of both implementation context (e.g., norms regarding HIV and HIV risk) and the users of the intervention [33]. Specifically, these findings reveal complex links between ‘sexual health literacy’ (which deeply connects ‘knowledge’ and sexual health practices to features of social contexts) and the extent to which PrEP is perceived of as a potential ‘replacement’ for condom use or other risk-reduction practices. We suggest research is needed to better characterize the rapidly evolving HIV intervention landscape and the social contexts within which the technical aspects of the HIV prevention ‘repertoire’ is unfolding. Furthermore, in light of the shifting intervention landscape in HIV (e.g., pharmaceutical interventions; the spread of routine testing; evolving gender ‘norms’), we were struck by the intensity with which study participants held up condom use as a “gold standard” risk-reduction practice. We also were struck by their deployment of stigmatizing discourse regarding ‘promiscuity’ (i.e., multiple sex partners). We suggest that contemporary public health messaging strategies about HIV prevention (even in a progressive and innovative context like Vancouver) need to be crafted to reflect the availability of an increasingly diverse range of HIV prevention tools and to reduce stigma.

The current study has several strengths and limitations. First, while the composition of our sample was intended to reflect the diversity of young men living in Vancouver, it is not intended to be ‘representative’. Second, as our study includes men that are at both ‘low’ and ‘high’ risk for HIV, we acknowledge that a different sample composition (e.g., one comprised solely of ‘low’ or only ‘high’ risk participants) may have resulted in a more polarized set of perceptions and expectations regarding PrEP and thus aligned more closely with previous acceptability research on PrEP. Nevertheless, including a diverse set of young men in our analysis helped us to uncover the influence of broad social norms and potentially stigmatizing discourse as serious implementation challenges for effective scaling up of PrEP in the Vancouver context.

## Conclusions

Based on the result of the current study, we suggest that pre-implementation strategies that concomitantly aim to improve knowledge about PrEP, while addressing associated social prejudices, may be key to its effective scale-up. Authentic engagement of a broad range of community-based stakeholders (e.g., HIV/AIDS NGOs) in the planning, implementation and monitoring of PrEP interventions will be essential [34]. As evidenced in the current study, moral opinions and prejudicial understandings comprise important aspects of the implementation context that may influence the feasibility, fidelity and equitable reach of interventions such as PrEP [35,36]. Research that examines moral preferences should not be practiced as simply ‘tallying’ public opinion (i.e., a descriptive endeavour), but rather as a participatory and transparent process that is used to inform and adapt interventions in ways that align with the key public health values and principles (e.g., health equity; solidarity) [35,37,38].
